# Nanocomposite microbeads made of recycled polylactic acid for the magnetic solid phase extraction of xenobiotics from human urine

**DOI:** 10.1007/s00604-024-06335-y

**Published:** 2024-04-09

**Authors:** Lorenzo Antonelli, Maria Chiara Frondaroli, Massimo Giuseppe De Cesaris, Nina Felli, Chiara Dal Bosco, Elena Lucci, Alessandra Gentili

**Affiliations:** Department of Chemistry, Sapienza University, P.Le Aldo Moro 5, 00185 Rome, Italy

**Keywords:** Solid phase extraction, Microbeads, Green analytical chemistry, Carbon nanomaterials, Recycled plastic, Polylactic acid

## Abstract

Nanocomposite microbeads (average diameter = 10–100 µm) were prepared by a microemulsion-solidification method and applied to the magnetic solid-phase extraction (m-SPE) of fourteen analytes, among pesticides, drugs, and hormones, from human urine samples. The microbeads, perfectly spherical in shape to maximize the surface contact with the analytes, were composed of magnetic nanoparticles dispersed in a polylactic acid (PLA) solid bulk, decorated with multi-walled carbon nanotubes (mPLA@MWCNTs). In particular, PLA was recovered from filters of smoked electronic cigarettes after an adequate cleaning protocol. A complete morphological characterization of the microbeads was performed via Fourier-transform infrared (FTIR) spectroscopy, UV–Vis spectroscopy, thermogravimetric and differential scanning calorimetry analysis (TGA and DSC), scanning electron microscopy (SEM) and X-ray diffraction analysis (XRD). The recovery study of the m-SPE procedure showed yields ≥ 64%, with the exception of 4-chloro-2-methylphenol (57%) at the lowest spike level (3 µg L^−1^). The method was validated according to the main FDA guidelines for the validation of bioanalytical methods. Using liquid chromatography-tandem mass spectrometry, precision and accuracy were below 11% and 15%, respectively, and detection limits of 0.1–1.8 µg L^−1^. Linearity was studied in the range of interest 1–15 µg L^−1^ with determination coefficients greater than 0.99. In light of the obtained results, the nanocomposite microbeads have proved to be a valid and sustainable alternative to traditional sorbents, offering good analytical standards and being synthetized from recycled plastic material. One of the main objectives of the current work is to provide an innovative and optimized procedure for the recycling of a plastic waste, to obtain a regular and reliable microstructure, whose application is here presented in the field of analytical chemistry. The simplicity and greenness of the method endows the procedure with a versatile applicability in different research and industrial fields.

**Graphical Abstract:**

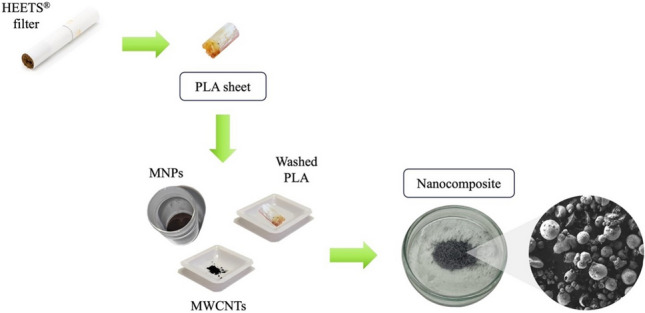

**Supplementary Information:**

The online version contains supplementary material available at 10.1007/s00604-024-06335-y.

## Introduction

Within Green Analytical Chemistry, one of the main objectives of today’s research is the quest for sorbent materials that, according to the 3rd principle of Green Sample Preparation (GSP), have characteristics of sustainability, reusability and renewability [1]. This tendency goes together with the miniaturization and automation of existing extraction techniques [2, 3] to reduce waste (4th principle), minimize sample, chemicals and materials (5th principle), and cut down energy consumption (8th principle) [1]. To have an idea of the current trends of microextraction innovative technique, framework that acts as a background of the presented research, a quite detailed table is reported in the Electronic Supplementary Material (Table S1). The use of new technologies to make such materials smaller can lead to significant operating advantages. Compared to conventional micro-sized sorbents, the nano-scaled ones exhibit wider specific surface area, improved sorption capacity, greater surface energy, higher diffusivity, and a more rapid achievement of adsorption equilibrium [4]. Nanostructured or nanocomposite materials are of special interest in sample preparation [5]. Two different approaches can be used to realize a nanocomposite [6]. The first one is a bottom-up approach, which involves the realization of a material starting from the single monomer up to a microstructure. The alternative is the top-down method, which begins with a pre-synthesized polymer and allows for reshaping it into a microscopic structure. The aspiration to recycle polymeric material from consumer sources is best served by a top-down approach [7].

Between 2016 and 2017, the World Economic Forum and the Ellen MacArthur Foundation launched the “New Plastic Economy” initiative [8] with the aim to promote a change in the use of plastics around three mainstays: redesign, reuse, and recycling. In 2018, the European Union declared in their new plastics strategy that 100% of plastics should either be reusable or recyclable by 2030 [9]. In this context, Analytical Chemistry may play its role, recycling plastics to prepare new materials for analytical applications. If within the sample preparation sector, it is common to use microparticle polymeric sorbents for solid phase extraction (SPE), there have been not many papers dealing with the preparation of sorbents from recycled plastic so far [10, 11]. In 2016, Psillakis et al. reported for the first time the use of low-density polyethylene plastic pellets as a low-cost and effective sorbent for extracting polycyclic aromatic hydrocarbons from environmental waters [10]. In 2017, Cárdenas et al. recycled polystyrene, whose degradation rate in the environment is very low, to synthesize magnetic nanocomposites for the dispersive micro-SPE of parabens from water samples [7].

The present article fits into this context by proposing the recycling of polylactic acid (PLA) into nanocomposite microbeads to extend the usable life of this plastic and to reduce the pollution generated by its littering [11]. The PLA used in this work was recovered from the filters of heated tobacco electronic cigarettes (HEETS®), which have skyrocketed in popularity especially among young people becoming one of the most diffuse solid wastes. PLA, which in HEETS® filters acts as a cooling agent for the aerosol created by heating tobacco [12], is a plastic-biopolymer that, in recent years, has attracted considerable attention thanks to its excellent properties of good workability and mechanical resistance [13]. The synthesis of the magnetic nanocomposite microbeads is based on a microemulsion-solidification method after a careful cleaning of the PLA-based filters. The technical advantage of the use of magnetic responsive materials in sample preparation applications is already well defined and reported in literature, both for solid and liquid phase extraction techniques [14, 15]. On this purpose, the use of magnetic nanoparticles (MNPs) is the recommended solution to guarantee a homogeneous distribution of the magnetic properties [16]. PLA itself did not show high affinity with the studied xenobiotics. To enhance the adsorption ability of the material, different active carbon materials were tested as coadjutants of the adsorption and components of the nanocomposite. As already reported, multi walled carbon nanotubes (MWCNTs) and graphene oxide (GO) have excellent properties for analytical applications but some drawbacks from the applicability point of view [17–19]. The strong interaction between different nanotubes or GO sheets is responsible for the aggregation of the material and a loose in terms of superficial area and extraction yields. The presented material aim is to use the bulk polymer (PLA) as a solid dispersant agent for carbonaceous active adsorbent, to avoid aggregation and to let MWCNTs and GO exploit their adsorption ability at full power, against the target analytes. The efficiency of the filter washing procedure as well as the synthesized nanocomposite microbeads were characterized via Fourier-transform infrared (FTIR) spectroscopy, UV–Vis spectroscopy, thermogravimetric and differential scanning calorimetry analysis (TGA and DSC), scanning electron microscopy (SEM) and X-ray diffraction analysis. Finally, the magnetic microbeads were evaluated for the magnetic-SPE (m-SPE) of fourteen model compounds (among pesticides, non-steroidal anti-inflammatory drugs, and hormones) from urine samples. The chosen xenobiotics are commonly found in urine of workers employed in the agricultural sector and selected as reference analytes for the evaluation of the adsorption performances of the synthetized material.

## Materials and methods

### Materials and reagents

PLA was recovered from filters of HEETS® cigarettes produced by one of the largest tobacco companies in the world. MWCNTs (length 6–13 nm, diameter 2.5–20 μm) and graphene oxide (GO; 15–20 sheets) were purchased from Merck Life Science S.r.l. (Milan, Italy).

A neodymium magnet from Atechmagnet (Beijing, China) was used as a magnetic lure.

Tetrahydrofuran (THF), isopropyl alcohol (≥ 99.5%), isopropyl acetate (≥ 99.6%), acetonitrile (≥ 99.9%), and sodium hydroxide (≥ 98%) were purchased from Merck Life Science S.r.l. (Milan, Italy). Absolute ethanol (≥ 99.5%) and hydrochloric acid (37.0%) were bought from VWR International (Radnor, USA). Formic acid (≥ 98%) was from Acros Organics B.V.B.A. (Waltham, USA). Milli-Q water was generated by the “Direct-Q® 3 UV System”, Merck KGaA (Darmstadt, Germany).

Iron (III) chloride (≥ 97.0%) and iron (II) sulphate (≥ 99.0%), the salts used to prepare MNPs were bought from Merck Life Science S.r.l. (Milan, Italy).

The standard used for the analytical procedure were: 4-chloro-2-methylphenol (≥ 98.0%), bensulfuron-methyl (≥ 98.0%), carprofen (≥ 98.0%), diclofenac (≥ 98.5%), diuron (≥ 98.0%), flamprop (≥ 95.0%), ibuprofen (≥ 98.0%), linuron (≥ 98.0%), malathion (≥ 98.0%), 4-(4-Chloro-2-methylphenoxy)butanoic acid (MCPB) (≥ 98.0%), methyl-testosterone (≥ 98.0%), 2-( ±)-(4-chloro-2-methyl)phenoxypropanoic acid (mecoprop; ≥ 98.0%), nimesulide (≥ 98.0%), progesterone (≥ 99.0%), creatinine (≥ 99.0%). All analytical standards were purchased from Merck Life Science S.r.l. (Milan, Italy), except for flamprop, purchased from Lab. Instruments S.R.L. (Bari, Italy), and MCPB and nimesulide, purchased from VWR International (Radnor, USA). The chemical structure, exact mass, IUPAC name, chemical classification, and agrochemical/pharmaceutical action of each analyte is reported in Table S2 in the Supplementary material. The individual standards were weighed using a precision analytical balance (Ohaus DV215CD Discovery semi-micro and analytical balance, 81/210 g capacity, 0.01/0.1 mg readability) and diluted in 1 mL of methanol (VWR International, Radnor, USA) to prepare stock solutions (1 mg mL^−1^). A stock composite standard solution of the 14 analytes was prepared at 100 µg L^−1^. Other working solutions and calibrators at different concentrations were prepared by diluting the stock composite standard solution with methanol. All solutions were stored at 4 °C.

### Urine samples

Urine samples, used as analyte-free matrices, were taken daily from healthy male volunteers, aged between 25 and 30 years, within our lab. A pool of urine from the different donors (~ 50 mL) was then subsampled to be used for the method optimization and validation. All urine samples were iced and stored at − 18 °C till their analysis.

### Synthesis of magnetic nanoparticles

The A. Avram et al. [20, 21] synthesis procedure was used to prepare magnetic nanoparticles having proven effective for controlling size and magnetic properties. For the detailed procedure, please refer to Electronic Supplementary Material (Section S.1).

### Synthesis of nanocomposite microbeads

Used-PLA filters (50 filters) were shredded and washed with 100 mL of hot ethanol at 50 °C for 30 min. After being used, ethanol was distilled and reused for a subsequent washing cycle. The cleaned PLA sheets were dried at room temperature for the next synthetic procedure, consisting in a microemulsion-solidification process. To this end, 57 mg of PLA was solubilized in 2 mL of THF to obtain a saturated solution. 2.8 mg of MWCNTs and 18 mg of MNPs was added to this organic solution, which was then transferred into a 10 mL glass vial (diameter: 2 cm) containing a magnetic bar on the bottom. The effect of alternating a stirring cycle (10 min at 600 rpm) with gentle heating (about 50 °C for 2 min) resulted in the production of a homogeneous dispersion of components. Five minutes in an ultrasonic bath favored the dispersion of MWCNTs, avoiding the formation of aggregates in solution.

**Fig. 1:**
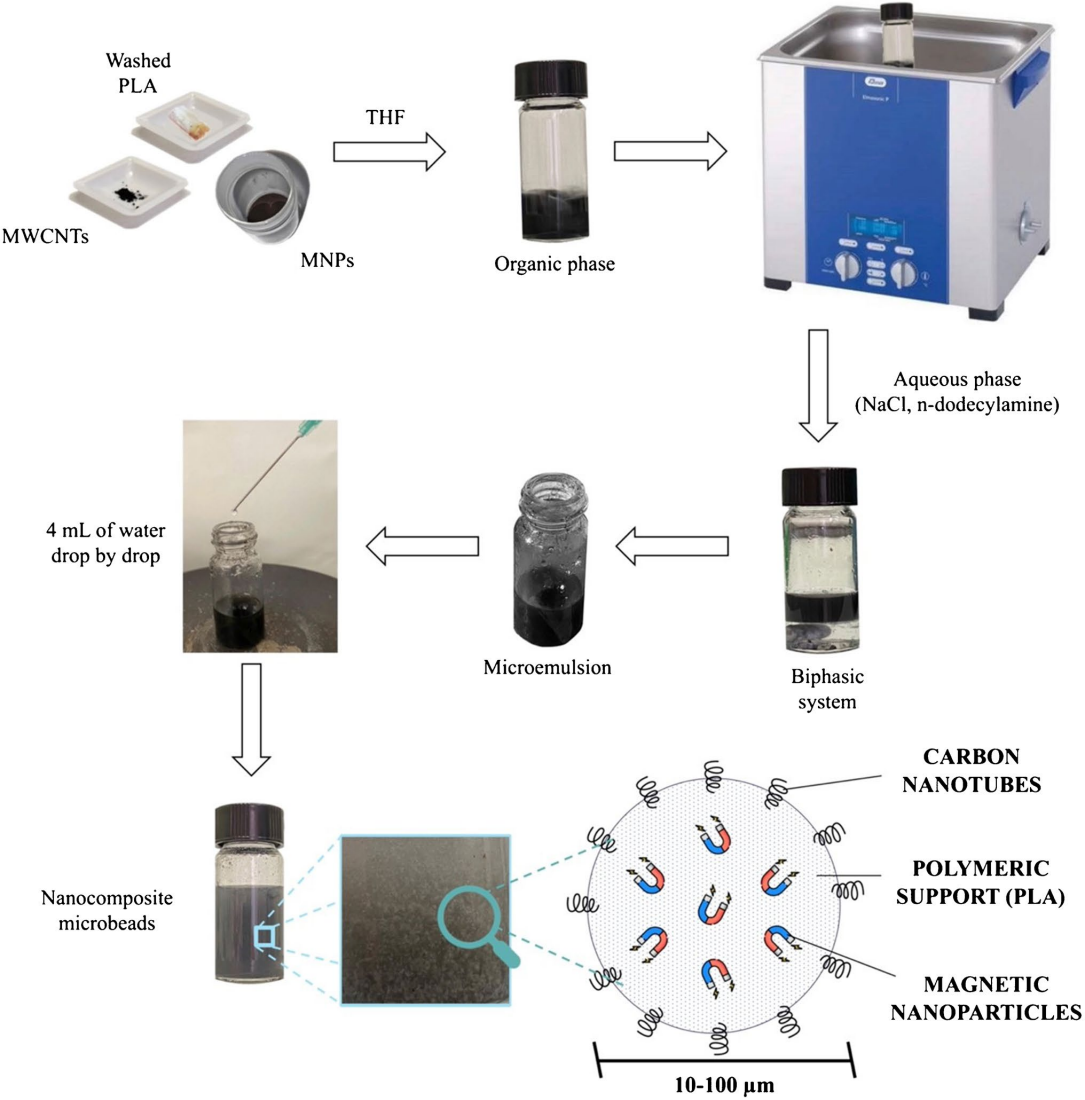
Representation of the synthetic strategy for the realization of mPLA@MWCNTs(5) nanocomposites and schematic image of the final material structure

Saturated NaCl aqueous solution and 2% w/v n-dodecylamine aqueous solution were prepared and mixed in a ratio 1:1 v/v. The resulting aqueous solution was mixed 1:1 v/v with the previous organic dispersion in another glass vial. The formation of a biphasic system was the result of the salting-out effect. The two-phases system was shaken (600 rpm) on a magnetic stirrer to form a microemulsion with microdroplets of the organic phase dispersed into the aqueous phase. To break down the emulsion and solidify PLA in the shape of microbeads, 4 mL pure water was added drop by drop. After removing the supernatant, the obtained nanocomposite was manually shaked three times with 5 mL of Milli-Q water to remove the salt and surfactant excess. After each washing, the supernatant was separated by magnetic capture of the microbeads. Finally, the composite was dried under nitrogen flow. The whole procedure takes less than 10 min, and the synthesis yield of the final material (mPLA@MWCNTs(5); see the “Selection and characterization of the most performant nanocomposite microbeads” section for the acronym explanation) is higher than 90% (in terms of final weight of microbeads with respect to the total weight of components). Figure 1 shows the cross-section of a magnetic microbead and provides a detailed scheme of the microbeads preparation procedure.

### Characterization of the magnetic nanocomposite microbeads

Several instrumental techniques were applied to check the efficiency of the filter cleaning procedure (ATR-FTIR and UV–Vis spectroscopy), the morphology of magnetic microbeads (SEM and XRD) and their stability (TGA and DSC).

The ATR-FTIR spectra were collected by using a Nicolet 6700 (Thermo Fisher Scientific, Waltham, USA) equipped with a Golden Gate single-reflection diamond with a resolution of 2 cm^−1^ and co-addition of 200 scans.

The UV–Vis analysis was conducted with a Model 760 spectrophotometer from PG Instrument Limited (Leicester, UK).

The TGA analysis was carried out with a Mettler TG 50 thermobalance (Mettler Toledo, Columbus, USA). 5 mg of a sample (PLA; microbeads) was placed in the platinum crucible and the analysis was performed under nitrogen flow, in the temperature range between 25 and 500 °C, with a heating rate of 10 °C min^−1^.

Differential Scanning Calorimetry (DSC) analysis of the composite device was performed by a Mettler TA-3000DSC apparatus. Thermograms were acquired at 10 °C min^−1^ in the + 25 to + 250 °C temperature range, under N_2_ flux.

The microbeads were also analyzed with an AURIGA model SEM (Carl Zeiss, Oberkochen, Germany, 0.5–30 keV, 10–10 mbar) to investigate their sizes and morphology.

X-ray diffraction (XRD) measurements were performed by a Malvern Panalytical X'Pert PRO apparatus (Cu Kα radiation, λ = 1.54184 Å) in an angle scan range (2θ) between 10 and 90 for a structural characterization of the composite. The peaks were identified through the instrumental spectral library.

### Extraction procedure

**Fig. 2:**
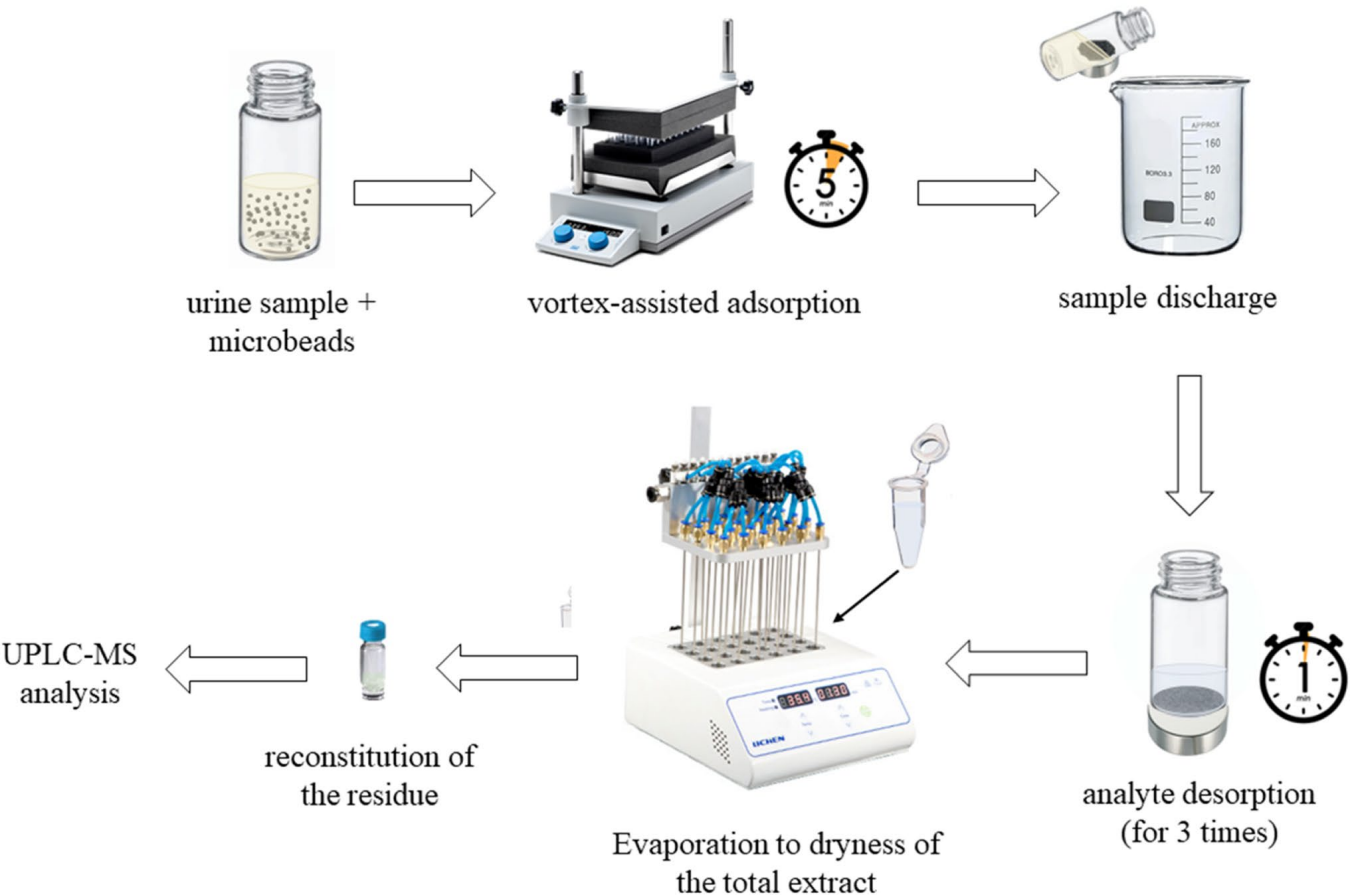
Schematic representation of the extraction procedure

A 0.6-mL aliquot of urine was diluted with 0.4 mL of Milli-Q water (V_T_ = 1 mL) and poured into a glass vial containing 15 mg of mPLA@MWCNTs(5). The analyte adsorption on the microbeads was assisted by mixing on a vortex-stirrer for 5 min. Then, the magnetic microbeads were recovered with a magnetic lure and the analytes were desorbed three times with 500 μL of methanol. The pooled extract (1.5 mL) was transferred to an Eppendorf tube and dried under nitrogen flow. The residue was reconstituted in 100 μL of mobile phase, consisting of a 1:1 (v/v) solution of acetonitrile and water; finally, 10 μL was injected in the chromatographic system. The entire analytical procedure is displayed in Fig. 2.

Analyte concentrations were normalized towards creatinine concentration as follows (Eq. (1)):

1 $${C}_{normalized}=\frac{{C}_{analyte }in\;\mu g\;{L}^{-1}}{{C}_{creatinine} in\;g\;{L}^{-1}}$$

### Creatinine determination

Creatinine concentration in urine reaches up to 0.4–3.0 g L^−1^ [22]. Owing to these high concentrations, 20 µL of urine was diluted with Milli-Q water in a 20-mL volumetric flask; then, a 2-μL volume was directly injected for the UPLC-MS analysis. Being the matrix effect negligible due to the high dilution ratio (1:1000), the concentration of creatinine in real samples was calculated by means of external calibration, building a calibration curve in solvent [23, 24].

### UPLC-MS/MS conditions

The analyte separation was performed using an ACQUITY UPLC H-Class PLUS® instrumentation from Waters Corporation (Milford, MA, USA). The column was an ACQUITY UPLC® BEH C_18_ (2.1 × 100 mm, 1.7 μm), purchased from Waters Corporation (Milford, MA, USA). The column was protected by a VanGuard Pre-Column® with the same stationary phase, 2.1 × 5 mm sized. The separation was carried out by using purified water (A) and acetonitrile (B), both acidified with 0.1% of formic acid, at a flow rate of 0.3 mL min^−1^. The elution was as follows: t_0_-t_10_ in isocratic mode at 50% B; t_10_ -t_30_ in linear gradient from 50 to 100% of B.

The mobile phase was entirely directed towards the Turbo V source, equipped with the electrospray probe, of a triple quadrupole mass spectrometer (API 4000 Qtrap from AB SCIEX, Foster City, CA, USA). The detection was performed in dual polarity mode (capillary voltage: + 5000 V in positive mode and − 4500 V in negative mode) by acquiring in Multiple Reaction Monitoring (MRM) and selecting two MRM transitions per analyte. Nitrogen in its purest state was supplied by a generator (Parker-Balston model 75A74, Haverhill, MA, USA) (nitrogen collision gas: 4 mTorr; nitrogen curtain gas: 5 L min^−1^) connected to a compressor (Jun-Air 4000-40 M, Bromsgrove, UK) (air nebulizer gas 2 L min^−1^; air drying gas at 450 °C and 20 L min^−1^).

To operate with a unit resolution, the full width at half maximum (FWHM) was set at 0.7 ± 0.1 m/z in each mass-resolving quadrupole.

**Fig. 3:**
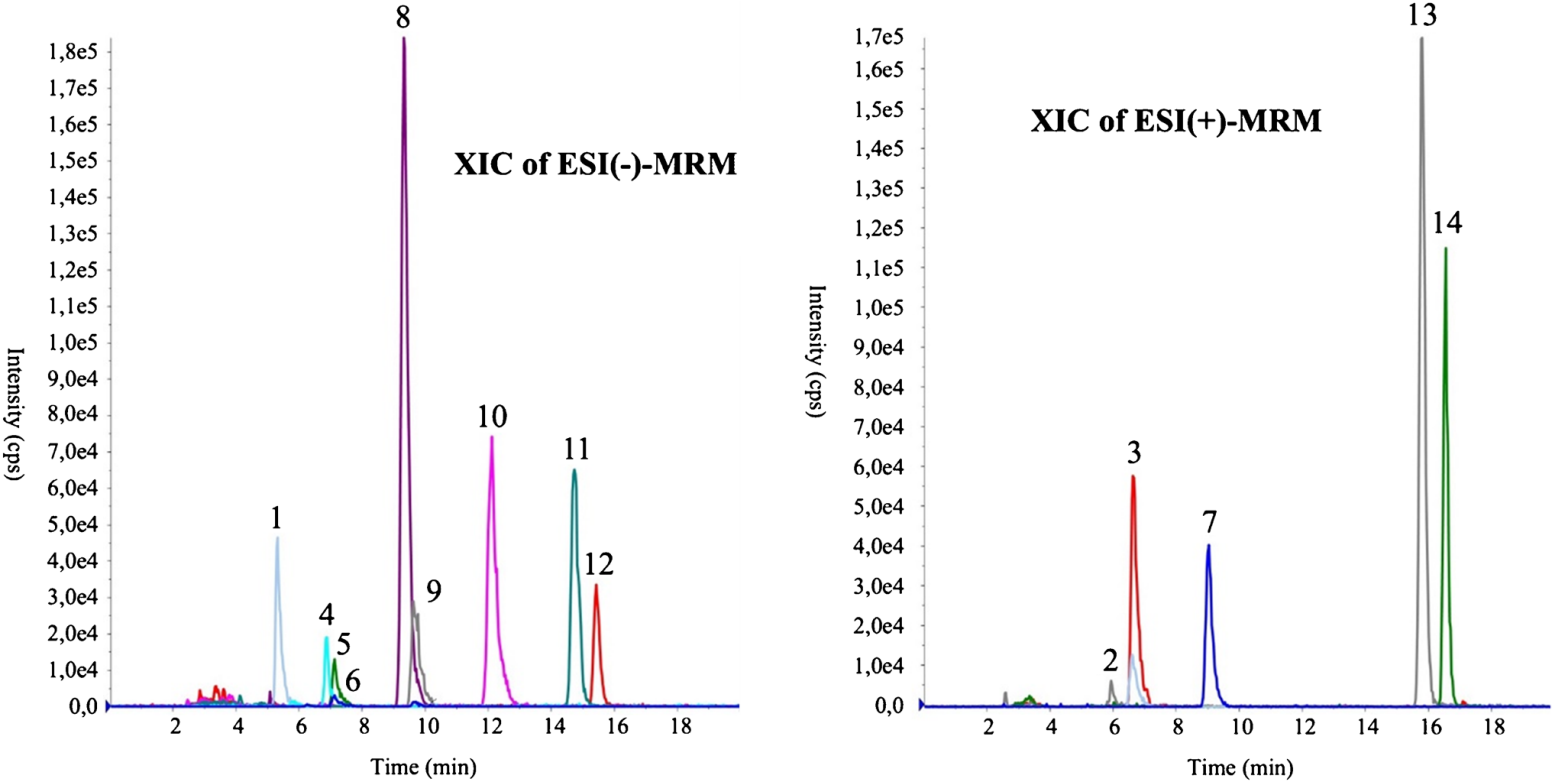
UPLC-MRM chromatograms in positive and negative polarity, under the optimized chromatographic conditions. The 14 analytes are separated in less than 18 min

The LC–MS parameters for each of the 14 analytes, selected in this study, are reported in Table 1. Figure 3 shows the UPLC-MRM chromatogram resulting from the injection of 5 µL of the working composite solution (0.5 ng injected).

**Table 1:** LC–MS parameters for the identification of the fourteen analytes under optimized conditions

Elution order	Compound	Retention time (min)	1st Transition (m/z) a	2nd Transition (m/z) a	Detection polarity ( +)/( −)
1	Diuron	5.31	230.9/149.7	230.9/185.8	−
2	Bensulfuron-methyl	6.60	411.1/149.1	411.1/182.1	+
3	Methyl-testosterone	6.64	303.2/109.2	303.2/97.1	+
4	Flamprop	6.87	320.0/121.0	320.0/248.0	−
5	4-chloro-2-methylphenol	7.12	141.0/104.9	141.0/76.8	−
6	Mecoprop	7.12	213.0/140.8	213.0/71.0	−
7	Linuron	9.03	249.0/182.1	249.0/159.9	+
8	Nimesulide	9.33	307.0/228.9	307.0/198.0	−
9	MCPB	9.62	227.1/140.9	227.1/104.9	−
10	Carprofen	12.11	272.0/228.0	272.0/212.8	−
11	Diclofenac	14.73	294.1/249.9	294.1/213.8	−
12	Ibuprofen	15.43	205.2/160.9	205.2/188.9	−
13	Malathion	15.79	331.0/127.1	331.0/285.0	+
14	Progesterone	16.55	315.2/109.1	315.2/97.1	+

^**a**^ The first MRM transition is the most intense one (quantifier), while the other is the second most intense (qualifier)

The software used to acquire and process the LC–MS data was Analyst 1.5.1.

Urinary creatinine, which is a chemical metabolism by-product whose excretion is not affected by urine flow [25], was determined to correct the analyte concentration in urine. The determination of creatinine was achieved in a separate run by using the same column and mobile phases in isocratic conditions, maintaining 65% of phase A for 5 min at the flow of 0.300 mL min^−1^. Retention time was 1.4 min and the monitored MRM transitions were 114.0/44.2 (qualifier) and 144.0/86.0 (quantifier).

### The method validation

The method was validated in matrix according to the FDA guidelines for the validation of bioanalytical methods [26]. Recovery, within-run and between-run precision and accuracy, limit of detention (LOD), lower limit of quantification (LLOQ), sensitivity, and linearity were the parameters evaluated. All calculations were performed by using with Microsoft Excel 2010 (Microsoft Corporation, Redmond, WA, USA).

## Results and discussion

### Optimization of the washing procedure to clean HEETS® filters

The nanocomposite microbeads were prepared by recycling thin sheets of PLA from HEETS® filters. The individual components of a HEETS® filter are shown in Fig. S1 of the Supplementary Material. The filter that comes into direct contact with the lips of the user is made up of cellulose acetate. To lower the vapor temperature, a cooling plug made of PLA is required. A ventilation chamber, in contact with the tobacco, consists of a cellulose acetate cylinder, with a hole in the middle. After the tobacco stick’s usage, the PLA film shows a change in color and consistency due to the high temperature of the steam and the deposition of low boiling products caught. Therefore, a preliminary washing procedure was introduced to return a polymer as similar as possible to the pristine PLA sheet. To this end, low toxic and low volatile solvents such as water, ethanol, isopropyl alcohol, and isopropyl acetate were tested to clean the material, keeping the properties of the polymer unchanged.

**Fig. 4:**
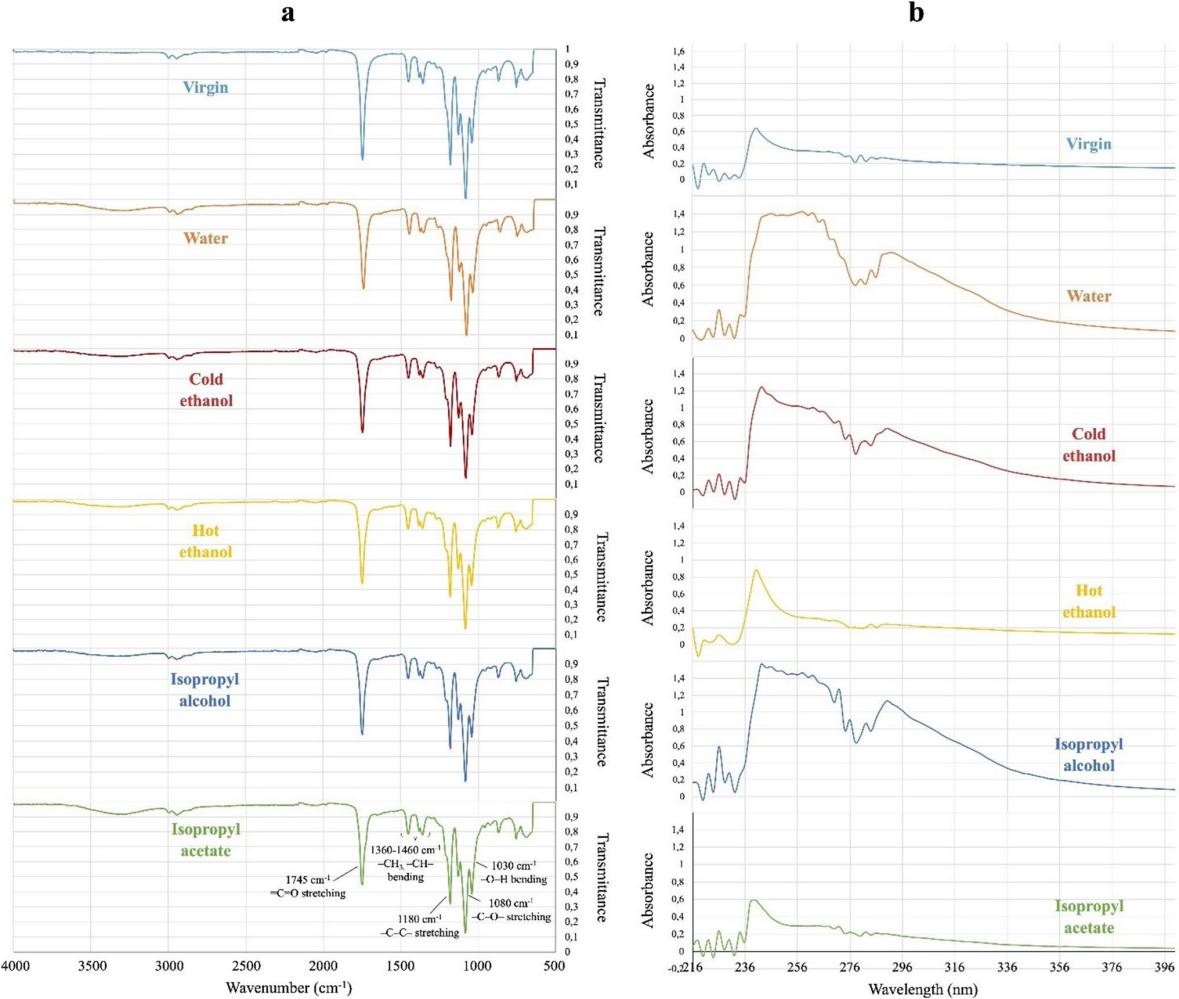
ATR-FTIR (**a**) and UV–Vis (**b**) spectra of the used-PLA filters: comparison among the spectral profiles obtained with different solvents as cleaning agents

About 12.50 g of dirty PLA, were immersed in 100 mL of a washing solvent, and magnetically stirred to promote optimal contact between the solvent and the polymer. The removal capability of a solvent system was evaluated by ATR-FTIR (Fig. 4a) and UV–Vis (Fig. 4b) spectroscopic analyses, comparing the spectra of PLA sheets treated under different conditions with the one from unused tobacco sticks.

A treated-PLA portion was put on the zinc selenide crystal of the ATR-FTIR instrument (Fig. S2a). The results indicate that hot ethanol and isopropyl acetate guarantee the highest degree of cleanliness preserving at the same polymeric materials. Hot ethanol was thus selected thanks to its lower toxicity, cost, and ease of recycling.

Before the UV–Vis analysis, the PLA sheets washed with the selected solvents were solubilized in 2 mL of tetrahydrofuran (Fig. S2b). The spectra in Fig. 4b, acquired for both the cleaned PLA sheets and the pristine one, show how the different solvents affected the polymer.

### Selection and characterization of the most performant nanocomposite microbeads

A characterization study on the synthesized microbeads was performed by ATR-FTIR, TGA, DSC, XRD and SEM analyses.

Six types of microbeads differing for carbon nanomaterials (MWCNTs, GO) and their quantity (5%, 10%, 20% of the polymeric weight) were synthesized. In what follows, we will refer to these materials using a common strategy of abbreviation. The bulk polymer is reported before the symbol “@”, the carbon nanomaterial is mentioned after the same symbol, and in round brackets there is its percentage with respect to the total weight of the polymer. The capital M at the beginning revers to the magnetic properties of the obtain materials (e.g. mPLA@GO(15) is the PLA-based material containing 15% w/w of GO).

Among the six types of microbeads, mPLA@GO(15) and mPLA@MWCNTs(5) provided the best performance when tested (15 mg of sorbent per test) to recover the 14 analytes from urine samples spiked at 1 μg L^−1^ (see Fig. S3a). Based on the observed trend, other nanocomposites were prepared: one with the 1.8% of MWCNTs (w/w) and another one with the 20% of GO (w/w). In the first case, a dramatic reduction in extraction yield was obtained, while in the second one, a structural failure of the nanocomposite material was observed, with the collapse of the spherical shape as an effect of the high quantity of GO. However, it was observed that the two materials show an opposite tendency to adsorption: for GO adsorption rates tend to increase as the percentage of active sorbent increases; on the other hand, for MWCNTs, an increase of the adsorption performances is registered with decreasing quantity of carbonaceous sorbent. This result can be explained in light of the better dispersion of MWCNTs in the PLA bulk at lower concentrations, that influences most the absolute recovery than the total quantity and availability of the carbonaceous active adsorbent. As already stated, the risk of the aggregation of CNTs is enhanced at higher concentrations and when an adequate dispersion in the polymeric support is not provided. On the other hand, since aggregation tendency is less significant for GO, greater extraction yields are provided by the devices that can guarantee an improved availability of active carbonaceous adsorption surface, namely the ones with higher concentrations of GO.

Both mPLA@GO(15) and mPLA@MWCNTs(5) were submitted to characterization. To confirm the preservation of the polymeric structure, clean PLA sheets were also analyzed.

The comparison of the ATR-FTIR spectra of carbonaceous nanocomposites with the one of the pure PLA is shown in Fig. S4a. A wide and weak band around 3000 cm^−1^ is due to the –OH stretching (free) of the PLA chain. Sharp bands, between 2940 and 3000 cm^−1^ are caused by the symmetric and asymmetric stretching of –CH– bonds. The carbonyl stretching (= C = O) is responsible for a sharp and intense band at 1760 cm^−1^. Two signals between 1360 and 1460 cm^−1^ are the result of the bending of –CH_3_ groups. Signals below 1300 cm^−1^ are much less informative and arise from the stretching of the –C-O– and C—O—C bond and bendings of the carbonyl and hydroxyl groups. Similar results and spectral evidence are reported in different previous works, here cited for comparison purposes [27, 28]. Spectroscopic profiles for the tested materials were almost completely superimposable and the major bands were similar for all the types of tested nanocomposites. The synthetic protocol does not result in an alteration of the bulk polymeric structure.

The Fig. S4b shows the TGA curves of mPLA@GO(15), mPLA@MWCNTs(5), and pristine PLA sheet. The thermogravimetric profiles are similar for all materials, which exhibit a single thermal transition, corresponding to a mass loss of 75%, in the same temperature range of PLA degradation, as reported in the literature [29]. Such results confirm the preservation of the polymer bulk composition in the nanocomposite microbeads.

The DSC analyses on the composite device (Fig. S5) shows an endothermic double peak between 150 and 162.5 °C associated with the temperature melting point I of PLA [30].

The characteristic double peak may be related to the complex structure of PLA where several crystalline domains coexist, as a result of the organization of different lengths polymer chains. The first peak would be relatable to the melting of smaller crystals or shorter polymer chains, while greater thermal energy is required for larger crystals melting [31, 32].

The XRD diffractogram, shown in Fig. S6 confirms the synthesis procedure is effective in the incorporation of magnetite and PLA into the composite. The most intense peak located at 14.8°, 16.4°, 19.2°, and 22.5° can be associated with the semicrystalline structure of PLA [33, 34] while the ones at 30.4°, 35.6°, and 43.2° can be related to iron oxide nanoparticles [35].

**Fig. 5:**
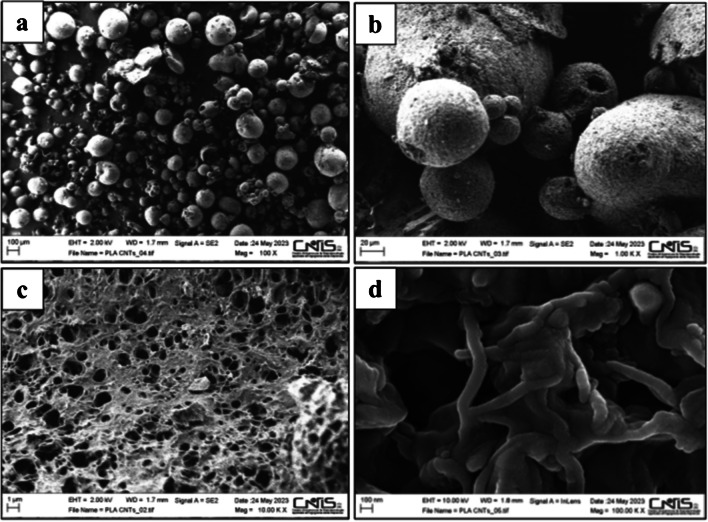
SEM images of mPLA@MWCNTs(5) at the following magnifications: 100X (**a**), 1.00 K X (**b**), 10.00 K X (**c**) and 100.00 K X (**d**)

Both nanocomposites were studied by means of SEM at different magnifications: (a) 100 X, to have an overlook of the obtained material; (b) 1.00 K X, to verify the spherical shape of the nanocomposite; (c) 10.00 K X, to observe the porous surface; (d) 100.00 K X, to observe the MWCNT nanostructure and the arrangement of the PLA filaments. From the images, it is possible to assess that the microbead diameters are dispersed in a narrow range of sizes, between 10 and 100 µm. From a comparison between both types of microbeads, mPLA@MWCNTs(5) seems to be more regular in shape and in the surface conformation. Figures 5a–d shows the images of mPLA@MWCNTs(5).

mPLA@MWCNTs(5) microbeads were thus selected for further steps of optimization considering it the best compromise between the synthesis greenness and the analytical performance.

### Optimization of the extraction procedure

The experiments to optimize the m-SPE procedure with mPLA@MWCNTs(5) were performed using 1-mL aliquots of diluted urine (spike level of urine before dilution with water was 1 μg L^−1^). To this end, a One-Variable-At-a-Time (OVAT) optimization approach (three replicates for each condition tested) was adopted.

The first parameter to be optimized was the amount of the sorbent: 10 mg, 15 mg, and 20 mg. Adsorption time and desorption time were kept at 30 min; for the analyte desorption was used a 2-mL volume of methanol. Recoveries obtained with 15 and 20 mg of microbeads were substantially unvaried (⁓77%), while 10 mg of mPLA@MWCNTs(5) were not sufficient to provide a complete extraction of the analytes from urine (61%) (see Fig. S3b). Although the dispersion of 15 mg and 20 mg leads to similar results, it was chosen to disperse the smaller amount in a greener perspective.

The following experiments, performed using 15 mg of mPLA@MWCNTs(5) were aimed at optimizing the adsorption time. To this end, a kinetic study was performed by evaluating the chromatographic area, averaged on all the analytes, at the following contact times: 5 min, 15 min, 1 h, 3 h and 6 h. The desorption step was carried out in a fixed contact time (30 min). Plotting the average chromatographic area against the adsorption time (see Fig. S7a), an asymptotic value is reached for all the analytes in less than 10 min. The results show that the adsorption rate of the analytes on the microbead surface is very fast. After the first 5 min of contact, no further significant adsorption was observed, due to the complete saturation of the available sites on the microbead surface. For this reason, it was decided to select a contact adsorption time of 5 min, as a compromise between processing time and extraction efficiency.

Once the adsorption time was selected, the desorption kinetic was investigated. The pretreatment procedure was repeated performing the desorption step with methanol (2 mL) at different contact times: 1 min, 2.5 min, 5 min and 10 min. The desorption curves are shown in Fig. S7b. The results prove that, even in this case, the kinetics are fast, and the plateau is easily reached within 1 min. Therefore, this time was selected to obtain the analyte desorption from the active sites of microbeads.

Finally, keeping unvaried the optimized parameters, it was evaluated type and volume of the desorption solvent (methanol, acetonitrile, and ethyl acetate). To this end, for each desorption solvent tested, four 0.5-mL fractions were collected and analyzed. The recovery yields showed that three 0.5 mL fractions of methanol allowed one to maximize the average recovery of the analytes (76%).

### Results of the method validation

Tables 2 and S3 summarize the figures of merit of the validated method (LOD, LLOQ, recovery precision and accuracy), while Tables S4 and S5 list the calculated linear regression parameters (slope, related to the method sensitivity, and intercept).

**Table 2:** Figures of merit of the 14 xenobiotics analyzed with the m-SPE-UPLC-MS/MS method proposed in this work. Recovery, precision and accuracy are reported for the lowest level of fortification (within three times the analyte LLOQ)

Compound	LOD (µg/L)	LLOQ (µg/L)	Recovery (%)	Precision (%)		Accuracy (%)	
				Within-run	Between-run	Within-run	Between-run
Diuron	0.4	0.8	64	5.0	5.8	13.5	14.0
Bensulfuron-methyl	0.6	0.9	76	7.2	9.0	8.5	9.2
Methyl-testosterone	0.5	0.9	68	7.6	7.9	10.0	10.6
Flamprop	0.6	1.0	72	6.8	7.4	10.2	10.8
4-Chloro-2-methylphenol	1.8	3.0	57	5.6	6.2	5.1	5.7
Mecoprop	1.3	2.2	86	9.0	10.6	9.9	10.5
Linuron	0.4	0.7	75	5.8	8.8	5.2	5.5
Nimesulide	0.1	0.3	69	9.0	10.7	5.4	6.0
MCPB	0.5	0.8	70	4.7	5.2	5.0	5.5
Carprofen	0.6	1.0	89	6.4	7.0	9.2	9.9
Diclofenac	0.2	0.4	100	5.5	6.3	5.4	6.0
Ibuprofen	0.5	0.8	70	5.5	5.8	5.1	5.5
Malathion	0.2	0.3	94	5.9	6.5	5.0	5.5
Progesterone	0.7	0.4	79	4.7	5.5	13.3	13.8

Matrix-matched calibration curves were prepared by analyzing 7 calibrators, spiked pre-extraction with the analytes in a dynamic linear range of interest from 1 to 15 μg L^−1^. Slope and intercept were estimated by means of the least-square method using the linear model y = a + b C; the error associated to a and b was estimated too. The determination coefficients *R*^2^ were found to be higher than 0.99, showing a good linear correlation.

LOD was calculated as the analyte concentration capable of providing a signal 3 times higher than the background noise (S = 3N). Therefore, 0.6-mL aliquots of urine were spiked with decreasing concentrations of the analytes, diluted with 0.4 mL of Milli-Q water (1 mL of diluted urine), and analyzed until a signal to noise ratio of about 3 was reached for each analyte. Likewise, LLOQ was calculated as the concentration generating a signal 5 times higher than the background noise (S = 5N) provided that precision and accuracy values were within 20%. Once established, LODs and LLOQs were confirmed by means of five independent replicates.

Recovery, precision and accuracy were estimated at the three different levels of fortification: 1 μg L^−1^ for all the analytes with the exception of 4-chloro-2-methylphenol and mecoprop, for which the spike level was set at 3 μg L^−1^ (i.e., within three times the analyte LLOQ; see Table 2); 6 μg L^−1^ (i.e. around 50% of the linear dynamic range; see Table S3); 15 μg L^−1^ (i.e. close to the upper limit of the calibration curve; see Table S3). Five replicates were performed at each level. Precision and accuracy provided *within-run* figures of merit when calculated on the same analytical session and *between-run* figures of merit when calculated on three different analytical sessions.

For each analyte, the recovery rates were calculated as (Eq. (2))

2 $$R\%=\frac{{C}_{measured}}{{C}^{*}}x100$$

where C* is the spike level applied and *C*_*measured*_ is the average concentration measured at a specific spike level. To calculate *C*_*measured*_, the average of the chromatographic areas obtained at each spike level (5 replicates) was interpolated in the post-extraction calibration curve (Table S5 shows the linear regression parameters; the linear dynamic range is the same as for the pre-extraction spiked curves). Recoveries, calculated at the different spike levels (see Table 2 for the lowest spike level and Table S3 for the other two ones), were greater than 64% with the exception of 4-chloro-2-methylphenol that shows a yield of 57% at the lowest spike level.

The precision was expressed in terms of relative standard deviation (RSD%). According to the FDA criteria, the RSD% should be ≤ 15%, with the only exception of the lowest fortification level (within 3 times LLOQ), for which an RSD ≤ 20% is also allowed. Tables 2 and S3 show that all RSD were lower than 15%.

The relative accuracy was evaluated as follows (Eq. (3))

3 $$Accuracy\%= \frac{{C}_{spiked}-{C}_{measured}}{{C}_{spiked}}x100$$

In this case, *C*_*measured*_ was calculated by interpolating the average of the chromatographic areas, obtained at each spike level, in the pre-extraction calibration line. As for precision, the accepted deviation for the accuracy evaluation is a maximum of 20% for the lowest level of fortification and 15% for all the others. Even in this case, the accuracy values are all lower than 15%, as it is shown in Table 2 and S3.

Finally, the enrichment factors (EF) were calculated according to the following equation:

4 $$EF=\frac{{C}_{analyte\;in\;the\;final\;extract}}{{C}_{analyte\;in\;the\;urine\;sample}}$$

The results were spanned between 3.42 and 6.00 depending on the analyte recovery.

### Selectivity of the method and adsorption mechanism conclusions

From a mechanistic point of view, the extraction performances of the synthetized material can be explained by the interactions MWCNTs are responsible for. The parts of a MWCNT that are available for the adsorption of analytes are the external surface and the groove areas. The internal part of the nanotube is mostly inaccessible, for the presence of coaxial graphene sheets, round-folded, to give rise to smaller diameter tubes. For this reason, molecular volume and geometrical characteristics of the analytes are not a discriminant parameter for the definition of the recovery yields [36]. Otherwise, carbon nanotubes are responsible for non-covalent interactions, in particular Van der Wals, π-stacking and electron-donor–acceptor interactions; chemical moieties and specific electron distribution on the analyte surface are discriminant parameters responsible for the distinct affinity of xenobiotics on MWCNTs. The highest the hydrophobicity of the analyte is, the highest the possibility of the molecule to participate in weak non-polar interactions with MWCNTs, resulting in an increasing of the adsorption on the material surface [37]. This theoretical tendency is confirmed from the experimental point of view for our 14 analytes: the evidence of a linear correlation between logP of the analytes and extraction yields of the material is displayed in Fig. S8 of the Supplementary Material. Despite the clear linear dependence, the analytes in the current study were chosen in a quite narrow range of polarity, with logP varying between 1.6 and 4.4. In light of a parallel study conducted on a higher number of contaminants on spiked water samples, it was possible to recognize the same strong dependence between analytes logP and recovery efficiency of the prepared material. Analytes with higher logP values result in higher affinities with mPLA@MWCNTs(5), showing enhanced recovery values. The same tendency was already reported in literature in a comprehensive study, by Zhao et al. [38], relatively to the single MWCNTs. From this evidence, we concluded that the PLA support does not play a relevant role in the adsorption mechanism of the composite material. As a confirmation, experimental analysis was performed with magnetic microspheres, obtained following the same showed procedure, but delating the addition of the active carbonaceous adsorbent (MWCNTs). The recovery yields, as expected, decreased to values below 5%, giving an experimental confirmation of the spectator’s role of PLA in the adsorption dynamics. As already stated, the polymeric bulk is otherwise fundamental to disperse MWCNTs and avoid the aggregations of the structures, providing, at the same time, a micrometric-sized material, easy to collect and to manipulate [39]. These conclusions, along with the extremely wide applicability of the device as it was presented, makes the present material a good extraction device for organic xenobiotics, in particular for the low-polarity ones, with logP higher than 3.

### Comparison with previous methods

Table S6 reports the main figures of merit (recovery, precision, LLOQ, EF, and analysis time) of the described procedure as well as those of previous methods developed to extract the same analytes from human, animal and synthetic urine samples [40–46]. As far as method limits are concerned, our method has comparable or lower (normalized) values like some of the chromatographic methods relying on MS detection [43–46]. All methods show low EFs with the exception of the procedures based on stir bar sorptive extraction (SBSE) [44] and cloud point extraction (CPE) [43]. Regarding recovery and precision, our method exhibits comparable performance than the others, with the difference that we evaluated these parameters applying lower spike levels (from 10 up to 5000 times), except the SBSE-GC–MS method for the determination of ibuprofen [44] whose evaluation was performed at 0.5 µg L^−1^ due to the dilution of the extract before the injection (see Table S6). Regarding extraction time, our procedure is rapid even if three out of the eight compared procedures (dispersive liquid–liquid microextraction (DLLME) [40], CPE [43], disposable pipette extraction (DPE) [45]) show time shorter than 10 min. Besides being more sensitive, our method is more sustainable due to the use of recycled PLA. At the best of our knowledge, the present method is the first one reporting a quantification method for flamprop in urine.

## Conclusion

The PLA-based nanocomposite described in this work is a sustainable product due to the intrinsic nature of PLA, a biodegradable polymer derived from a renewable natural resource such as corn. The nanocomposite is simple to prepare and exhibits a perfect compatibility between PLA and the other components (MWCNTs/GO and magnetic nanoparticles). The micrometric size and reactivity to magnetic fields greatly simplify the extraction procedure, avoiding the use a of a centrifuge and reducing analysis time. The properties of the composite and the ease of application allowed us to obtain good analytical performance in terms of recoveries, precision and accuracy. The nanocomposite could also be applied to extract pollutants from environmental waters as well as for water remediation purposes.

Although PLA is biodegraded in the environment by enzymes and bacteria into CO_2_, water and humus, its high production to replace part of petroleum-based plastics is leading to a large amount of waste PLA accumulation due to its slow degradation rate [47]. Thus, the extension of PLA’s lifetime through recycling is a convenient solution with environmental and economic benefits because PLA is also an expensive polymer, more than conventional plastics such as polyethylene. Under this point of view, this work offers a contribute proposing a strategy of PLA recycling and a new use of such a polymer.

### Supplementary Information

Below is the link to the electronic supplementary material.

Supplementary file1 (DOCX 1390 KB)

## Data Availability

All data generated or analysed during this study are included in this published article and its supplementary information files.
