# Supplementary material for: Nanocomposite microbeads made of recycled polylactic acid for the magnetic solid phase extraction of xenobiotics from human urine
Source: Mikrochim Acta. 2024 Apr 9;191(5):251. doi: 10.1007/s00604-024-06335-y (PMC11001671; doi:10.1007/s00604-024-06335-y)
Supplement: Supplementary file 1 — Supplementary file1 (DOCX 1390 KB) [file 604_2024_6335_MOESM1_ESM.docx]

**Supplementary Material**

**Nanocomposite microbeads made of recycled polylactic acid for the magnetic solid phase extraction of xenobiotics from human urine**

Lorenzo Antonelli^a^, Maria Chiara Frondaroli^a^, Massimo Giuseppe De Cesaris^a^, Nina Felli^a^, Chiara Dal Bosco^a^, Elena Lucci^a^, Alessandra Gentili^a*^

^a^ Department of Chemistry, Sapienza University, P.le Aldo Moro 5, 00185, Rome, Italy

* To whom correspondence should be addressed. Phone: +39-06-49693230

E-mail address: [alessandra.gentili@uniroma1.it](mailto:alessandra.gentili@uniroma1.it)

**Table S1** The table reports some of the newly proposed microextraction techniques, from 2020 to 2024

| **Web searching keywords** | **Research paper name** | **Year** | **Method** | **Adsorbent material** | **Analytes of interest** | **Matrix** | **Reference** |
| --- | --- | --- | --- | --- | --- | --- | --- |
| nanocomposite microbeads microextraction | Using Fe_3_O_4_-graphene oxide-modified chitosan with melamine magnetic nanocomposite in the removal and magnetic dispersive solid-phase microextraction of Cr (VI) ion in aquatic samples | 2024 | Magnetic dispersive solid-phase microextraction | Magnetic melamine‐functionalized chitosan-modified graphene oxide (MCMGO) nanocomposite | Cr (VI) ion | Water | [1] |
| nanocomposite microbeads microextraction | Magnetic Luffa@metal-organic frameworks (MOF-199) nanocomposite for the solid phase microextraction of some metal ions at trace levels from food and water samples | 2023 | Solid phase microextraction | Magnetic Luffa@MOF-199 nanocomposite | Ni(II), Pb(II), Cr(III) and Cd(II) | Water | [2] |
| nanocomposite microbeads microextraction | Determination of selected pesticides by GC-FID after CNO/MOF  nanocomposites-based dispersive solid phase extraction coupled with  liquid microextraction | 2023 | Dispersive solid phase extraction (DSPE) | CNO-ZIF67 nanocomposite | Pesticides | Vegetables | [3] |
| nanocomposite microbeads microextraction | Covalent organic framework in situ grown on Fe_3_O_4_ hollow microspheres for stir bar sorptive‐dispersive microextraction of triazole pesticides | 2023 | Stir bar sorptive-dispersive microextraction | TFPB‐BD/Fe_3_O_4_ nanocomposite | Triazole pesticides | Fruit, vegetables | [4] |
| nanocomposite microbeads microextraction | Application of ZnS/S/S-RGO three-component nanocomposites in dispersive solid-phase microextraction coupled with ion mobility spectrometry for ultra-trace determination of multiclass pesticides | 2022 | Dispersive solid-phase microextraction | ZnS/S/S-RGO nanocomposite | Multiclass pesticides | Well water, agricultural wastewater, soil, and rice | [5] |
| nanocomposite microbeads microextraction | Application of Fe_3_O_4_@TbBd nanobeads in microextraction by packed sorbent (MEPS) for determination of BTEXs biomarkers by HPLC–UV in urine samples | 2022 | Microextraction packed sorbent  (MEPS) | Fe_3_O_4_@TbBd nanobeads | BTEXs biomarkers | Urine | [6] |
| nanocomposite microbeads microextraction | Novel polyphenol/graphene nanocomposite for solid-phase microextraction of bisphenol A and bisphenol B leached from plastic containers | 2021 | Solid-phase microextraction | Polyphenol (PPh) and graphene oxide  nanosheets (GONSs) nanocomposite | Bisphenol A and bisphenol B | Plastic | [7] |
| nanocomposite microbeads microextraction | Polyaniline-coated core-shell silica microspheres-based dispersive-solid phase extraction for detection of benzophenone-type UV filters in environmental water samples | 2021 | Dispersive-solid phase extraction (DSPE) | CSMS@PANI nanocomposite microspheres | Benzophenone-type UV filters | Water | [8] |
| nanocomposite microbeads microextraction | Core-shell structured Fe_2_O_3_/CeO_2_@MnO_2_ microspheres with abundant surface oxygen for sensitive solid-phase microextraction of polycyclic aromatic hydrocarbons from water | 2021 | Solid-phase microextraction | Fe_2_O_3_/CeO_2_@MnO_2_ microspheres | Polycyclic aromatic hydrocarbons | Water | [9] |
| nanocomposite microbeads microextraction | Sono-synthesized Fe_3_O_4_–GO–NH_2_ nanocomposite for highly efficient ultrasound-assisted magnetic dispersive solid-phase microextraction of hazardous dye Congo red from water samples | 2021 | Ultrasound-assisted magnetic dispersive solid-phase microextraction (UA-MDSPME) | Fe_3_O_4_–GO– NH_2_ nanocomposite | Congo red dye | Water | [10] |
| nanocomposite microbeads microextraction | An Ag_2_S@ZnS-coated fiber for efficient, long-life solid-phase microextraction of polycyclic aromatic hydrocarbons in water | 2020 | Solid-phase microextraction | Ag_2_S@ZnS-coated fiber | Polycyclic aromatic hydrocarbons | Water | [11] |
| nanocomposite microbeads microextraction | Decoration of Fe_3_O_4_@SiO_2_@ZnO as a high performance nanosorbent on a stir bar microextraction device for preconcentration and determination of cadmium in real water samples | 2020 | Stir bar microextraction | Fe_3_O_4_@SiO_2_@ZnO core–shell nanosorbent | Cadmium | Water | [12] |
| nanocomposite microbeads microextraction | Optimization by response surface methodology of a dispersive magnetic solid phase extraction exploiting magnetic graphene nanocomposite coupled with UHPLC-PDA for simultaneous determination of new oral anticoagulants (NAOs) in human plasma | 2020 | Dispersive magnetic solid phase extraction | Reduced graphene@ Fe_3_O_4_ nanocomposite | Novel oral anticoagulants (NOAs) | Human plasma | [13] |
| nanocomposite microbeads microextraction | Magnetic solid-phase extraction of sulfonamide antibiotics in water and animal-derived food samples using core-shell magnetite and molybdenum disulfide nanocomposite adsorbent | 2020 | Magnetic solid-phase extraction | Fe_3_O_4_@MoS_2_ nanocomposite | Sulfonamide antibiotics | Water, food | [14] |
| nanocomposite microbeads microextraction | Graphene oxide-Fe_3_O_4_ nanocomposite magnetic solid phase extraction followed by UHPLC-MS/MS for highly sensitive determination of eight psychoactive drugs in urine samples | 2020 | Magnetic solid phase extraction | GO- Fe_3_O_4_ nanocomposite | Eight psychoactive drugs | Urine | [15] |

[1] Bagheri V, Naseri A, Sajedi-Amin S, Soylak M, Zhang Z (2024) Using Fe_3_O_4_-graphene oxide-modified chitosan with melamine magnetic nanocomposite in the removal and magnetic dispersive solid-phase microextraction of Cr (VI) ion in aquatic samples. Chem. Pap. 78:381–396. https://doi.org/10.1007/s11696-023-03096-5.

[2] Ahmed HEH, Soylak M (2023). Magnetic Luffa@metal-organic frameworks (MOF-199) nanocomposite for the solid phase microextraction of some metal ions at trace levels from food and water samples. J. Food Compos. Anal. 121:105396-105407. https://doi.org/10.1016/j.jfca.2023.105396.

[3] Abbasalizadeh A, Ghalkhani M, Marzi Khosrowshahi E, Mazani A, Hosseini A, Sohouli E, Ahmadi F (2023) Determination of selected pesticides by GC-FID after CNO/MOF nanocomposites-based dispersive solid phase extraction coupled with liquid microextraction. Diam. Relat. Mater. 137:110087-110097. https://doi.org/10.1016/j.diamond.2023.110087.

[4] Wang YX, Shen XF, Feng YW, Pang YH (2023) Covalent organic framework in situ grown on Fe_3_O_4_ hollow microspheres for stir bar sorptive-dispersive microextraction of triazole pesticides. Microchim. Acta. 190:34-42. https://doi.org/10.1007/s00604-022-05613-x.

[5] Rahmani S, Aibaghi B (2022) Application of ZnS/S/S-RGO three-component nanocomposites in dispersive solid-phase microextraction coupled with ion mobility spectrometry for ultra-trace determination of multiclass pesticides. Microchim. Acta. 189:9-18. https://doi.org/10.1007/s00604-021-05116-1.

[6] Kurd N, Bahrami A, Afkhami A, Shahna FG, Assari MJ, Farhadian M (2022) Application of Fe_3_O_4_@TbBd nanobeads in microextraction by packed sorbent (MEPS) for determination of BTEXs biomarkers by HPLC–UV in urine samples. J Chromatogr. B 1197:123197-12406. https://doi.org/10.1016/j.jchromb.2022.123197.

[7] Behzadi M (2021) Novel polyphenol/graphene nanocomposite for solid-phase microextraction of bisphenol A and bisphenol B leached from plastic containers. Sens. Actuators A: Phys. 321:112599-112606. https://doi.org/10.1016/j.sna.2021.112599. https://doi.org/10.1016/j.sna.2021.112599.

[8] Wang A, Hu L, Liu J, Tian M, Yang L (2021) Polyaniline-coated core-shell silica microspheres-based dispersive-solid phase extraction for detection of benzophenone-type UV filters in environmental water samples. Environ. Adv. 3:100037-100045. https://doi.org/10.1016/j.envadv.2021.100037.

[9] Xu S, Dong P, Qin M, Liu H, Long A, Chen C, Feng S, Wu H (2021) Core-shell structured Fe_2_O_3_/CeO_2_@MnO_2_ microspheres with abundant surface oxygen for sensitive solid-phase microextraction of polycyclic aromatic hydrocarbons from water. Microchim. Acta 188:337-346. https://doi.org/10.1007/s00604-021-05004-8.

[10] Sricharoen P, Chanthai S, Lamaiphan N, Sakaew C, Limchoowong N, Nuengmatcha P, Oh WC (2021) Sono-synthesized Fe_3_O_4_–GO–NH_2_ nanocomposite for highly efficient ultrasound-assisted magnetic dispersive solid-phase microextraction of hazardous dye Congo red from water samples. J. Korean Ceram. Soc. 58(2):201–211. https://doi.org/10.1007/s43207-020-00089-y.

[11] Liu H, Fan H, Wang X, Dang S, Gu A (2020) An Ag_2_S@ZnS-coated fiber for efficient, long-life solid-phase microextraction of polycyclic aromatic hydrocarbons in water. J Sep. Sci. 43:3646–3654. https://doi.org/10.1002/jssc.202000282.

[12] Banihashemi M, Dalali N, Sehati N, Farajmand B (2020) Decoration of Fe_3_O_4_@SiO_2_@ZnO as a high performance nanosorbent on a stir bar microextraction device for preconcentration and determination of cadmium in real water samples. Microchem. J. 154:104599-104607. https://doi.org/10.1016/j.microc.2020.104599.

[13] Ferrone V, Todaro S, Carlucci M, Fontana A, Ventrella A, Carlucci G, Milanetti E (2020) Optimization by response surface methodology of a dispersive magnetic solid phase extraction exploiting magnetic graphene nanocomposite coupled with UHPLC-PDA for simultaneous determination of new oral anticoagulants (NAOs) in human plasma. J. Pharm. Biomed. Anal. 179:112992-113001. https://doi.org/10.1016/j.jpba.2019.112992.

[14] Zhao Y, Wu R, Yu H, Li J, Liu L, Wang S, Chen X, Chan D (2020) Magnetic solid-phase extraction of sulfonamide antibiotics in water and animal-derived food samples using core-shell magnetite and molybdenum disulfide nanocomposite adsorbent. J. Chromatogr. A 1610:460543-460550. https://doi.org/10.1016/j.chroma.2019.460543.

[15] Lu Q, Guo H, Zhang Y, Tang X, Lei W, Qi R, Chu J, Li D, Zhao Q (2020) Graphene oxide-Fe_3_O_4_ nanocomposite magnetic solid phase extraction followed by UHPLC-MS/MS for highly sensitive determination of eight psychoactive drugs in urine samples. Talanta 206:120202-120210. https://doi.org/10.1016/j.talanta.2019.120212.

**Table S2** The table shows the chemical structure of each pesticide, and lists: the exact mass, IUPAC name, chemical classification, and agrochemical/pharmaceutical action

| **Common name** | **Structure** | **Exact mass**  **(u)** | **IUPAC name** | **Chemical classification** | **Agrochemical/Pharmaceutical action** | **Ref.** |
| --- | --- | --- | --- | --- | --- | --- |
| Diuron | 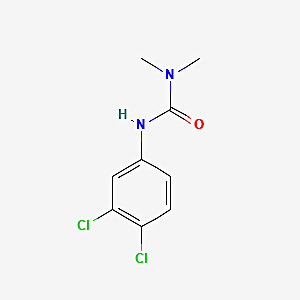 | 232.0170 | 3-(3,4-dichlorophenyl)-1,1-dimethylurea | Phenylurea derivatives | Herbicides | [1] |
| Bensulfuron-Me | 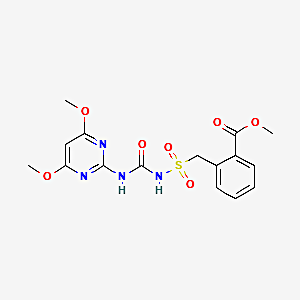 | 410.0896 | methyl 2-[(4,6-dimethoxypyrimidin-2-yl)carbamoylsulfamoylmethyl] benzoate | Sulfonylurea derivatives | Herbicides | [2] |
| Me-Testosterone | 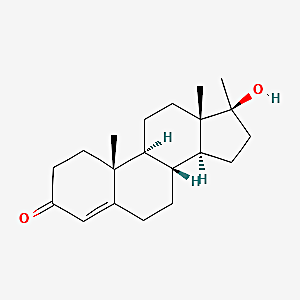 | 302.2245 | (8R,9S,10R,13S,14S,17S)-17-hydroxy-10,13,17-trimethyl-2,6,7,8,9,11,12,14,15,16-decahydro-1H-cyclopenta[a]phenanthren-3-one | Hydroxy steroids | Hormone | [3] |
| Flamprop | 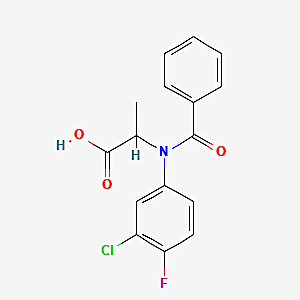 | 321.0568 | 2-(N-benzoyl-3-chloro-4-fluoroanilino)propanoic acid | Benzamides | Herbicides | [4] |
| 4-chloro-2-methylphenol | 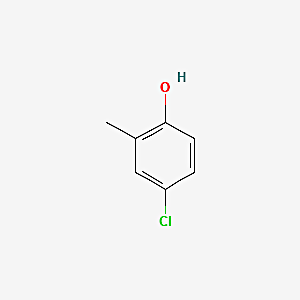 | 142.0185 | 4-chloro-2-methylphenol | Chlorophenols | Herbicides metabolite | [5] |
| Mecoprop | 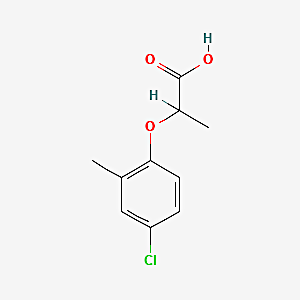 | 214.0397 | 2-(4-chloro-2-methylphenoxy)propanoic acid | Phenoxyacetates | Herbicide | [6] |
| Linuron | 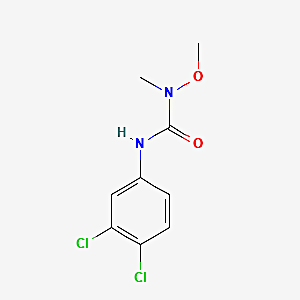 | 248.0119 | 3-(3,4-dichlorophenyl)-1-methoxy-1-methylurea | Phenylurea Compounds | Herbicides | [7] |
| Nimesulide | 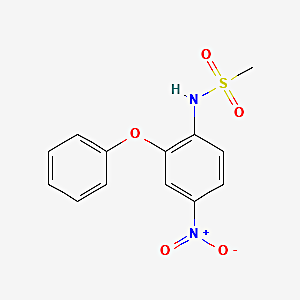 | 308.0467 | N-(4-nitro-2-phenoxyphenyl)methanesulfonamide | Sulfonamides | Nonsteroidal Anti-inflammatory Drug | [8] |
| MCPB | 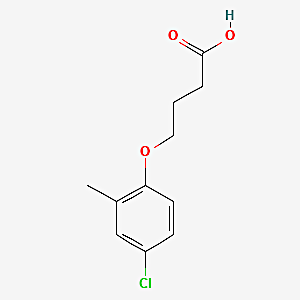 | 228.0553 | 4-(4-chloro-2-methylphenoxy)butanoic acid | Phenoxybutirrates | Herbicide | [9] |
| Carprofen | 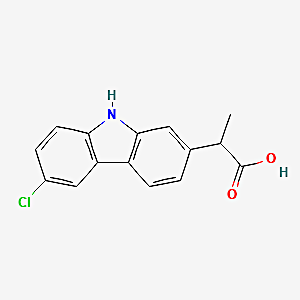 | 273.0557 | 2-(6-chloro-9H-carbazol-2-yl)propanoic acid | Carbazoles | Nonsteroidal Anti-inflammatory Drug | [10] |
| Diclofenac | 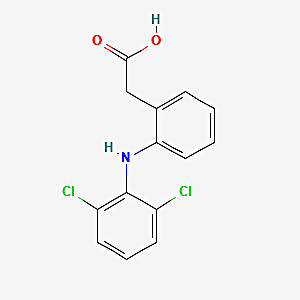 | 295.0167 | 2-[2-(2,6-dichloroanilino)phenyl]acetic acid | Phenylacetates | Enzyme Inhibitor | [11] |
| Ibuprofen | 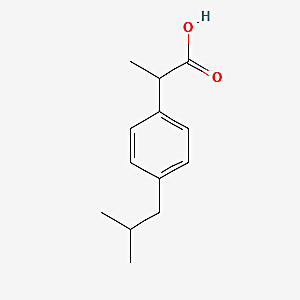 | 206.1307 | 2-[4-(2-methylpropyl) phenyl]propanoic acid | Phenylpropionates | Nonsteroidal Anti-inflammatory Drug | [12] |
| Malathion | 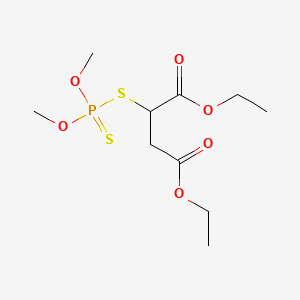 | 375.0485 | diethyl 2-dimethoxy phosphinothioyl sulfanylbutanedioate | Aryloxyphenoxypropionic esters | Herbicide | [13] |
| Progesterone | 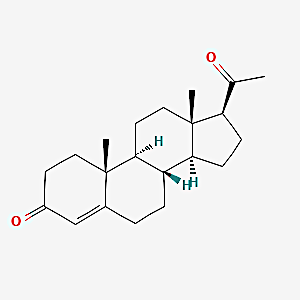 | 314.2246 | (8S,9S,10R,13S,14S,17S)-17-acetyl-10,13-dimethyl-1,2,6,7,8,9,11,12,14,15,16,17-dodecahydrocyclopenta[a]phenanthren-3-one | C21-Steroids | Hormone | [14] |

[1] *PubChem [Internet]. Bethesda (MD): National Library of Medicine (US), National Center for Biotechnology Information; 2004-. PubChem Compound Summary for CID 3120, Diuron; [cited 2023 Dec. 1]. Available from:* [*https://pubchem.ncbi.nlm.nih.gov/compound/Diuron*](https://pubchem.ncbi.nlm.nih.gov/compound/Diuron)

[2] *PubChem [Internet]. Bethesda (MD): National Library of Medicine (US), National Center for Biotechnology Information; 2004-. PubChem Substance Record for SID 318040044, 2-[[[[[(4,6-dimethoxy-2-pyrimidinyl)amino]carbonyl]amino]sulfonyl]methyl]benzoic acid methyl ester, Source: iChemical Technology USA Inc; [cited 2023 Dec. 1]. Available from: https://pubchem.ncbi.nlm.nih.gov/substance/318040044*

[3] *PubChem [Internet]. Bethesda (MD): National Library of Medicine (US), National Center for Biotechnology Information; 2004-. PubChem Compound Summary for CID 6010, Methyltestosterone; [cited 2023 Dec. 1]. Available from:* [*https://pubchem.ncbi.nlm.nih.gov/compound/Methyltestosterone*](https://pubchem.ncbi.nlm.nih.gov/compound/Methyltestosterone)

[4] *PubChem [Internet]. Bethesda (MD): National Library of Medicine (US), National Center for Biotechnology Information; 2004-. PubChem Compound Summary for CID 42807, Flamprop; [cited 2023 Dec. 1]. Available from: https://pubchem.ncbi.nlm.nih.gov/compound/Flamprop*

[5] *PubChem [Internet]. Bethesda (MD): National Library of Medicine (US), National Center for Biotechnology Information; 2004-. PubChem Compound Summary for CID 14855, 4-Chloro-2-methylphenol; [cited 2023 Dec. 1]. Available from:* [*https://pubchem.ncbi.nlm.nih.gov/compound/4-Chloro-2-methylphenol*](https://pubchem.ncbi.nlm.nih.gov/compound/4-Chloro-2-methylphenol)

[6] *PubChem [Internet]. Bethesda (MD): National Library of Medicine (US), National Center for Biotechnology Information; 2004-. PubChem Compound Summary for CID 7153, Mecoprop; [cited 2023 Dec. 1]. Available from: https://pubchem.ncbi.nlm.nih.gov/compound/Mecoprop*

[7] *PubChem [Internet]. Bethesda (MD): National Library of Medicine (US), National Center for Biotechnology Information; 2004-. PubChem Compound Summary for CID 9502, Linuron; [cited 2023 Dec. 1]. Available from: https://pubchem.ncbi.nlm.nih.gov/compound/Linuron*

[8] *PubChem [Internet]. Bethesda (MD): National Library of Medicine (US), National Center for Biotechnology Information; 2004-. PubChem Compound Summary for CID 4495, Nimesulide; [cited 2023 Dec. 1]. Available from: https://pubchem.ncbi.nlm.nih.gov/compound/Nimesulide*

[9] *PubChem [Internet]. Bethesda (MD): National Library of Medicine (US), National Center for Biotechnology Information; 2004-. PubChem Compound Summary for CID 7207, 4-(4-Chloro-2-methylphenoxy)butanoic acid; [cited 2023 Dec. 1]. Available from: https://pubchem.ncbi.nlm.nih.gov/compound/4-_4-Chloro-2-methylphenoxy_butanoic-acid*

[10] *PubChem [Internet]. Bethesda (MD): National Library of Medicine (US), National Center for Biotechnology Information; 2004-. PubChem Compound Summary for CID 2581, Carprofen; [cited 2023 Dec. 1]. Available from: https://pubchem.ncbi.nlm.nih.gov/compound/Carprofen*

[11] *PubChem [Internet]. Bethesda (MD): National Library of Medicine (US), National Center for Biotechnology Information; 2004-. PubChem Compound Summary for CID 3033, Diclofenac; [cited 2023 Dec. 1]. Available from: https://pubchem.ncbi.nlm.nih.gov/compound/Diclofenac*

[12] *PubChem [Internet]. Bethesda (MD): National Library of Medicine (US), National Center for Biotechnology Information; 2004-. PubChem Compound Summary for CID 3672, Ibuprofen; [cited 2023 Dec. 1]. Available from: https://pubchem.ncbi.nlm.nih.gov/compound/Ibuprofen*

[13] *PubChem [Internet]. Bethesda (MD): National Library of Medicine (US), National Center for Biotechnology Information; 2004-. PubChem Compound Summary for CID 4004, Malathion; [cited 2023 Dec. 1]. Available from: https://pubchem.ncbi.nlm.nih.gov/compound/Malathion*

[14] *PubChem [Internet]. Bethesda (MD): National Library of Medicine (US), National Center for Biotechnology Information; 2004-. PubChem Compound Summary for CID 5994, Progesterone; [cited 2023 Dec. 1]. Available from:* [*https://pubchem.ncbi.nlm.nih.gov/compound/Progesterone*](https://pubchem.ncbi.nlm.nih.gov/compound/Progesterone)

***S.1 Synthesis of magnetic nanoparticles***

To prepare MNPs, 1.352 g of FeCl_3_∙6H_2_O and 0.695 g of FeSO_4_∙7H_2_O (molar ratio 2:1) were dissolved in 200 mL of a 0.5 M HCl solution. After the drop-by-drop addition of a 1.25 M NaOH solution, a black precipitate of MNPs was immediately formed. The addition was stopped when a neutral pH was reached. The solid residue was then washed five times with 10 mL of Milli-Q water, every time by centrifuging and removing the washing water. After this procedure, the same cleaning step was realized with ethanol (10 mL). The dried solid residue was pounded in a glass mortar and the resulting powder stored in a weighing bottle.

**Fig. S1** Representation of the cross-section of a HEETS^®^ filter

**Fig. S2** Procedure for preparing the used-PLA filter to the ATR-FTIR (**a**) and UV-Vis (**b**) spectroscopic analysis

**a**

**b**


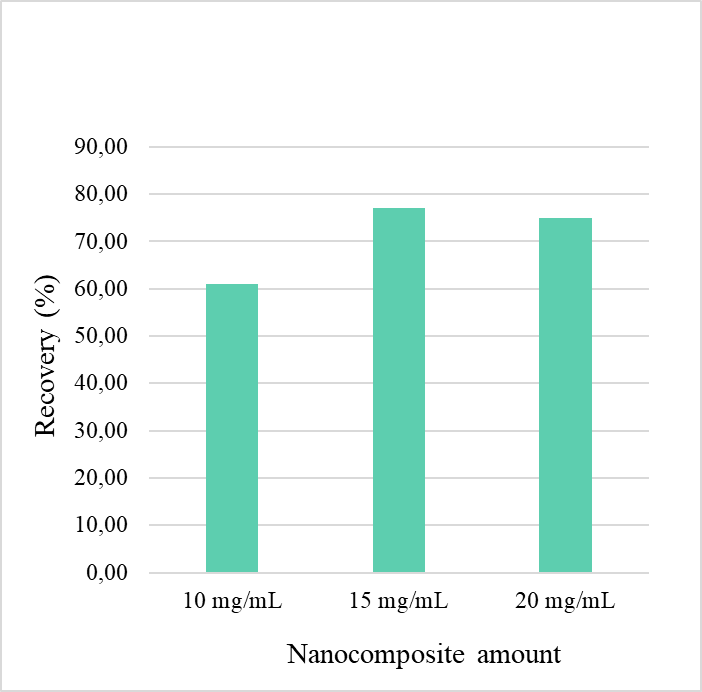

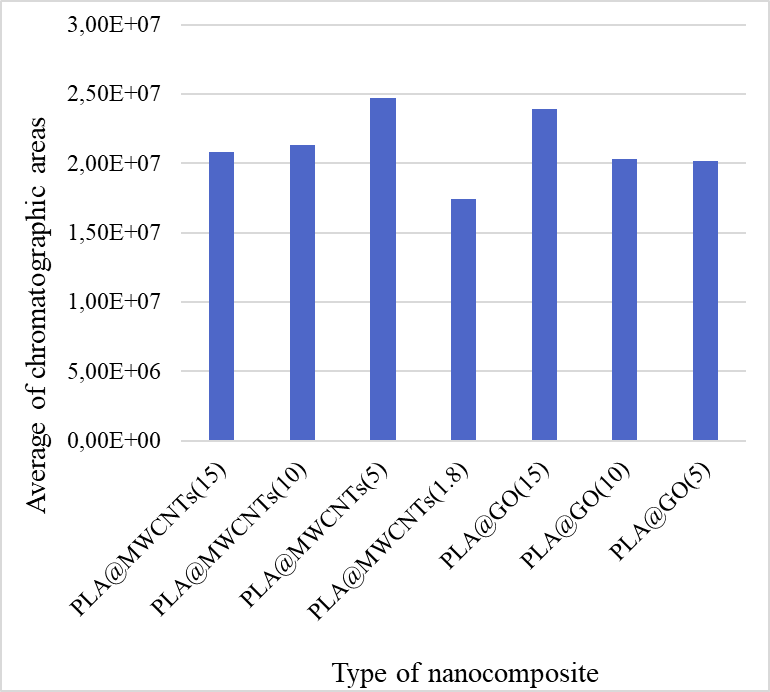


**Fig. S3** In **a**, the performance of the six types of microbeads was evaluated comparing the average chromatographic areas of all the analytes in the final extracts, achieved with the different materials. PLA@MWCNTs(5) and PLA@GO(15) registered the best efficiency in analytes recovery. In **b**, nanocomposite amount of PLA@MWCNTs(5) in 1 mL of spiked matrix solution is tested. By comparison among three reasonable conditions, the 15 mg/mL batch proves to be the best solution


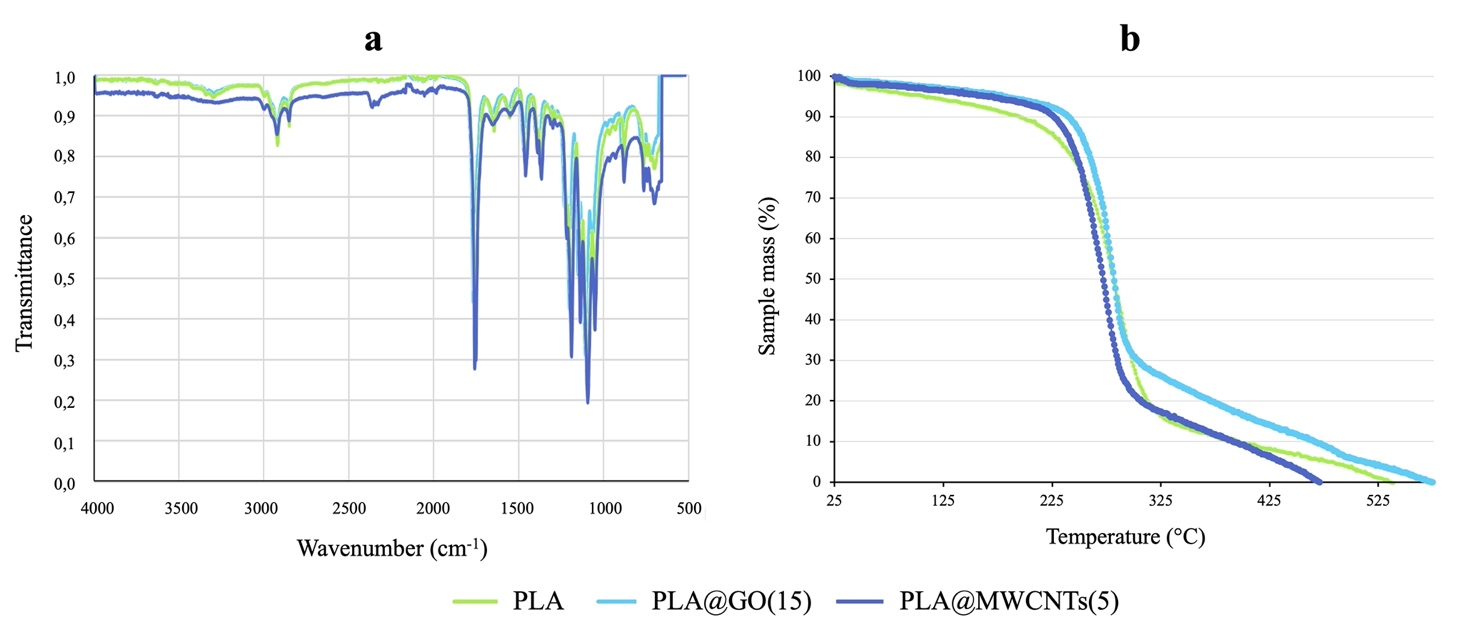


**Fig. S4** ATR-FTIR (**a**) and Thermogravimetric (**b**) analysis of pure PLA, PLA@GO(15) and PLA@MWCNTs(5) nanocomposites

**Fig. S5** Thermogram DSC of the composite device in which are visible the double endothermic peak of PLA associated with the melting of the polymer

**Fig. S6** Diffractogram of the composite device in with are visible the scattering pattern of PLA (orange triangles) and iron oxide (green triangles)


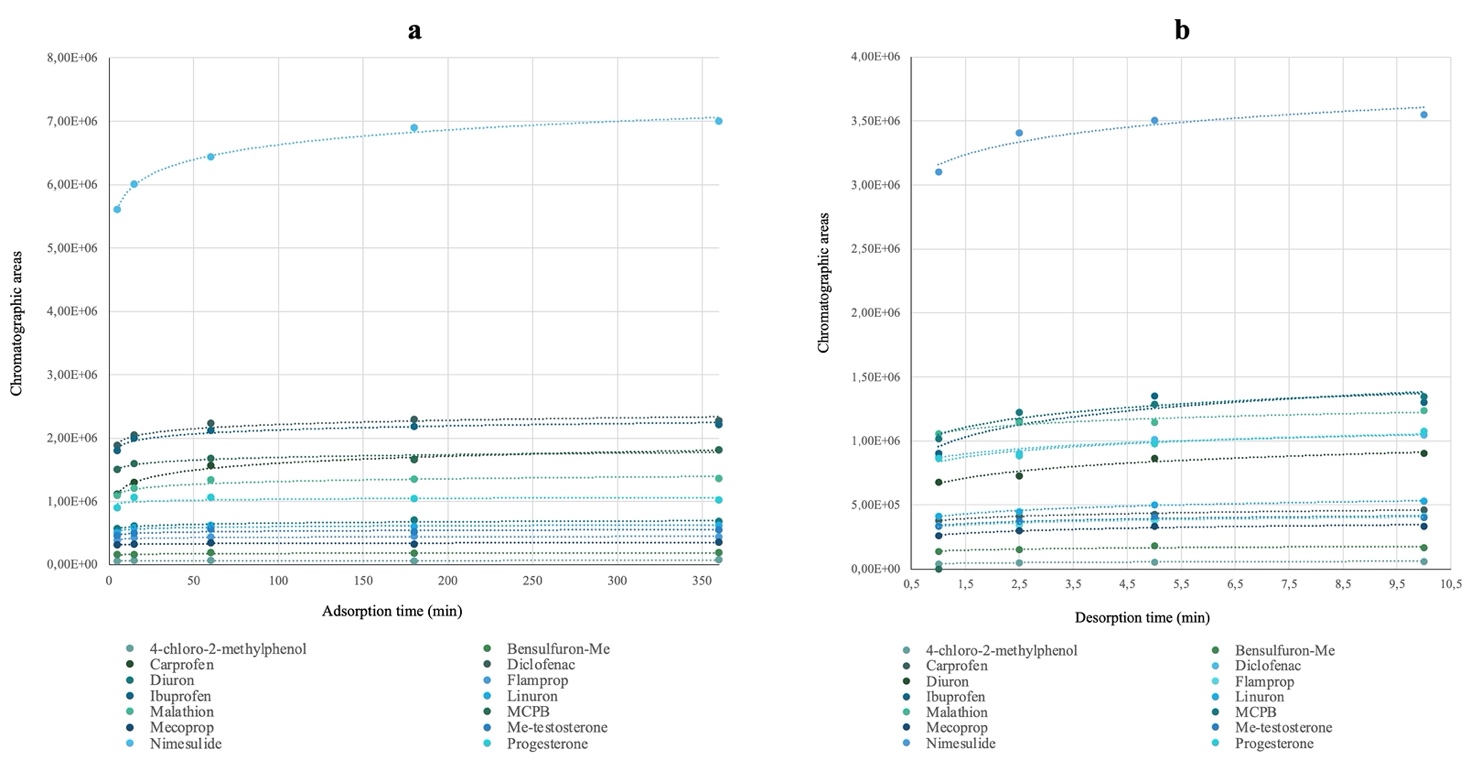


**Fig. S7** Kinetic experiments elucidate the time needed for the equilibrium to be reached in both adsorption (**a**) and desorption (**b**) steps. The adsorption equilibrium is reached in less than 5 minutes for most of the analytes. Otherwise, 1 minute is enough to consider concluded the desorption step for each involved analyte

**Table S3** Recovery, precision and accuracy of the 14 analytes analyzed with the m-SPE-UPLC-MRM method proposed in this work for the middle (6 μg L^-1^) and the highest spike levels (15 μg L^-1^)

| **Compound** | Spike level = 6 μg L^-1^ | | | | | Spike level = 15 μg L^-1^ | | | | |
| --- | --- | --- | --- | --- | --- | --- | --- | --- | --- | --- |
|  | **Recovery**  **(%)** | **Precision**  **(%)** | | **Accuracy**  **(%)** | | **Recovery**  **(%)** | **Precision**  **(%)** | | **Accuracy**  **(%)** | |
|  |  | **Within-run** | **Between-run** | **Within-run** | **Between-run** |  | **Within-run** | **Between-run** | **Within-run** | **Between-run** |
| Diuron | 70 | 3.0 | 5.2 | 13.0 | 13.4 | 74 | 2.0 | 5.0 | 12.8 | 13.0 |
| Bensulfuron-methyl | 80 | 6.8 | 8.8 | 8.0 | 8.9 | 80 | 6.5 | 8.0 | 8.0 | 8.6 |
| Methyl-testosterone | 70 | 6.6 | 7.0 | 10.0 | 10.2 | 72 | 6.6 | 7.2 | 9.8 | 10.0 |
| Flamprop | 75 | 6.2 | 6.4 | 10.0 | 10.4 | 76 | 6.0 | 6.4 | 9.8 | 10.0 |
| 4-Chloro-2-methylphenol | 64 | 3.6 | 5.2 | 5.1 | 5.5 | 65 | 3.0 | 5.0 | 5.0 | 5.4 |
| Mecoprop | 89 | 7.5 | 9.9 | 9.8 | 10.2 | 91 | 7.0 | 10.0 | 9.5 | 9.8 |
| Linuron | 80 | 3.8 | 5.0 | 5.0 | 5.5 | 80 | 3.5 | 4.6 | 5.0 | 5.2 |
| Nimesulide | 72 | 7.9 | 9.8 | 5.4 | 5.8 | 71 | 7.9 | 9.6 | 5.5 | 5.8 |
| MCPB | 77 | 3.7 | 4.2 | 5.0 | 5.5 | 78 | 2.9 | 4.0 | 5.0 | 5.4 |
| Carprofen | 93 | 5.6 | 5.9 | 9.0 | 9.4 | 93 | 5.0 | 5.2 | 8.7 | 9.0 |
| Diclofenac | 100 | 4.1 | 5.2 | 5.2 | 6.0 | 100 | 4.0 | 5.2 | 5.0 | 5.8 |
| Ibuprofen | 76 | 3.5 | 4.8 | 5.0 | 5.6 | 78 | 3.5 | 4.6 | 5.0 | 5.6 |
| Malathion | 95 | 4.7 | 6.8 | 5.0 | 5.4 | 95 | 4.6 | 6.6 | 4.8 | 5.2 |
| Progesterone | 82 | 3.7 | 3.7 | 13.0 | 13.5 | 84 | 2.7 | 3.2 | 12.5 | 12.9 |

**Table S4** Linear regression parameters for the calibration curves built spiking

the urine aliquots pre-extraction with the analytes (mean of six independent

analyses)

| **Compound** | **b ± S_b_t_(0.05;6)_**  (x 10^2^) | **a ± S_a_t_(0.05;6)_**  (x 10^2^) | **R^2^** |
| --- | --- | --- | --- |
|  |  |  |  |
| Diuron | 274.9 ± 6.9 | 35.4 ± 0.7 | 0.9904 |
| Bensulfuron-methyl | 92.2 ± 2.4 | 3.6 ± 0.2 | 0.9955 |
| Methyl-testosterone | 312.0 ± 9.4 | 48.2 ± 1.7 | 0.9902 |
| Flamprop | 98.1 ± 2.8 | 4.4 ± 0.3 | 0.9925 |
| 4-Chloro-2-methylphenol | 20.20 ± 0.46 | 2.30 ± 0.57 | 0.9938 |
| Mecoprop | 133.2 ± 4.0 | 21.3 ± 0.9 | 0.9903 |
| Linuron | 257.3 ± 7.5 | 5.60 ± 0.15 | 0.9930 |
| Nimesulide | 977 ± 20 | 144.8 ± 2.9 | 0.9927 |
| MCPB | 156.6 ± 3.9 | 30.4 ± 1.1 | 0.9904 |
| Carprofen | 199.7 ± 6.4 | 49.0 ± 1.3 | 0.9937 |
| Diclofenac | 563.8 ± 13.5 | 34.7 ± 0.66 | 0.9958 |
| Ibuprofen | 192.9 ± 6.4 | 40.7 ± 1.3 | 0.9938 |
| Malathion | 825 ± 19 | 26.48 ± 0.79 | 0.9907 |
| Progesterone | 500 ± 14 | 34.60 ± 0.69 | 0.9910 |

**Table S5** Linear regression parameters for the calibration curves built spiking the urine aliquots post-extraction with the analytes (mean of six independent analyses)

| **Compound** | **b ± S_b_t_(0.05;6)_**  (x 10^-2^) | **a ± S_a_t_(0.05;6)_**  (x 10^-2^) | **R^2^** |
| --- | --- | --- | --- |
|  |  |  |  |
| Diuron | 432.1 ± 8.7 | 1.66 ± 0.03 | 0.9907 |
| Bensulfuron-methyl | 121.5 ± 3.5 | 12.91 ± 0.39 | 0.9902 |
| Methyl-testosterone | 456 ± 10 | 93.20 ± 0.28 | 0.9960 |
| Flamprop | 136.2 ± 4.1 | 23.95 ± 0.72 | 0.9910 |
| 4-Chloro-2-methylphenol | 37.36 ± 0.93 | 11.55 ± 0.35 | 0.9903 |
| Mecoprop | 154.6 ± 3.9 | 15.04 ± 0.48 | 0.9950 |
| Linuron | 345.5 ± 9.0 | 1.301 ± 0.033 | 0.9977 |
| Nimesulide | 2103 ± 50 | 343 ± 10 | 0.9908 |
| MCPB | 436 ± 14 | 90.3 ± 2.9 | 0.9982 |
| Carprofen | 550 ± 15 | 79.0 ± 2.5 | 0.9905 |
| Diclofenac | 560 ± 18 | 119.4 ± 3.6 | 0.9943 |
| Ibuprofen | 276.2 ± 8.8 | 142.5 ± 4.7 | 0.9938 |
| Malathion | 875 ± 26 | 256.1 ±7.7 | 0.9799 |
| Progesterone | 632 ± 14 | 35.13 ± 0.70 | 0.9905 |

**Fig. S8** In the presented graph logP of analytes is plotted against the recoveries obtained with the presented analytical procedure. A general linear trend is shown by the data, displaying the occurrence of a linear dependence between the considered variables

**Table S6** Comparison with other methods aimed at determining the analytes selected in this study in urine samples

| **Common name** | **Method** | **Recovery** | **Precision** | **Enrichment factor** | **Method limits**  (µgL^-1^) | **Extraction time**  (min) | **Ref.** |
| --- | --- | --- | --- | --- | --- | --- | --- |
|  |  | (%)  (spike levels) | (RSD%)  (spike levels) |  |  |  |  |
| Diuron, Diclofenac | DLLME^a^- HPLC-DAD | 75-120  (25-250 µg L^-1^ ) | 8-21  (25-250 µg L^-1^) | ~13-16 | 10.0 (LOQ) | ~10 | [1] |
| Mecoprop, MCPB | SPE-Capillary LC–UV | 66-100  (25-150 µg L^-1^) | 1-5  (25-150 µg L^-1^ ) | - | 18-19 (LOQ) | ~30 | [2] |
| Linuron | QuEChERS-UHPLC-MS | 94-101  (10-50 µg L^-1^) | 4-9  (10-50 µg L^-1^) | ~0.5  (extract dilution) | 13.1 (LOQ) | >30 | [3] |
| Nimesulide | CPE^b^-HPLC–MS | 95.6-107.3  (67-5000 µg L^-1^) | 0.04-13  (67-5000 µg L^-1^) | ~55 | 67 (LOQ) | ~10 | [4] |
| Ibuprofen | SBSE^c^-GC-MS | 97.2-100.6  (0.5-5 µg L^-1^) | 4.37-4.57  (0.5-5 µg L^-1^) | ~245 | 0.53 (LOQ) | ~40 | [5] |
| Malathion | DPE-GC-MS | 71-87  (10-70 µg L^-1^) | 4.7-6.8  (10-70 µg L^-1^) | ~4 | 5.0 (LOQ) | 5 | [6] |
| Progesterone | LLE-UHPLC-MS | 94  (20-2000 µg L^-1^) | 7  (20-2000 µg L^-1^) | ~5 | 0.98 (LOQ) | 60 | [7] |
| Diuron, Diclofenac, Mecoprop, MCPB, Linuron, Nimesulide, Ibuprofen, Malathion, Progesterone | m-SPE-UHPLC-MS | 64-100  (1-15 µg L^-1^) | 2.0-10.7  (1-15 µg L^-1^) | ~6 | 0.3-2.2 (LLOQ) | 15 | This work |

^a^ DLLME = dispersive liquid–liquid microextraction; ^b^ CPE = Cloud point extraction; ^c^ SBSE = Stir bar sorptive extraction; ^d^ DPE = Disposable pipette extraction

[1] C. Will, E. Omena, G. Corazza, G. Bernardi, J. Merib, E. Carasek (2020). Expanding the applicability of magnetic ionic liquids for multiclass determination in biological matrices based on dispersive liquid–liquid microextraction and HPLC with diode array detector analysis. J. Sep. Sci., 43(13), 2657-2665. https://doi.org/10.1002/jssc.202000143.

[2] N. Rosales-Conrado, M. E. León-González, L. V. Pérez-Arribas, L. M. Polo-Díez (2008). Multiresidue determination of chlorophenoxy acid herbicides in human urine samples by use of solid-phase extraction and capillary LC–UV detection. Anal. Bioanal. Chem., 390, 759-768. https://doi.org/10.1007/s00216-007-1701-5

[3] C. Sweeney, Y. Park, J. S. Kim (2019). Comparison of sample preparation approaches and validation of an extraction method for nitrosatable pesticides and metabolites in human serum and urine analyzed by liquid chromatography-Orbital ion trap mass spectrometry, J. Chromatogr. A 1603 83–91. https://doi.org/10.1016/j.chroma.2019.06.065.

[4] O. G. Makukha, L. A. Ivashchenko, O. A. Zaporozhets, Volodymyr O. Doroschuk (2019). Cloud point extraction combined with HPLC-MS for the determination of nimesulide in biological samples, Chem. Zvesti. 73, 693–699. https://doi.org/10.1007/s11696-018-0618-0.

[5] P. Mohammadi, M. Masrournia, Z. Es’haghi, M. Pordel (2021). Hollow fiber coated Fe3O4@Maleamic acid-functionalized graphene oxide as a sorbent for stir bar sorptive extraction of ibuprofen, aspirin, and venlafaxine in human urine samples before determining by gas chromatography-mass spectrometry, J. Iran. Chem. Soc. 18 2249–2259. https://doi.org/10.1007/s13738-021-02185-0.

[6] A. Luiz Oenning, J. Merib, E. Carasek (2018). An effective and high-throughput analytical methodology for pesticide screening in human urine by disposable pipette extraction and gas chromatography-mass spectrometry. J. Chromatogr. B, 1092, 459-465. https://doi.org/10.1016/j.jchromb.2018.06.047.

[7] Y. Zhou, Z. Cai (2020). Determination of hormones in human urine by ultra-high-performance liquid chromatography/triple-quadrupole mass spectrometry, Rapid Communications in Mass Spectrom. 34 e8583. https://doi.org/10.1002/RCM.8583.
